# Affect and Cognition in Attitude Formation toward Familiar and Unfamiliar Attitude Objects

**DOI:** 10.1371/journal.pone.0141790

**Published:** 2015-10-30

**Authors:** Roxanne I. van Giesen, Arnout R. H. Fischer, Heleen van Dijk, Hans C. M. van Trijp

**Affiliations:** Marketing and Consumer Behaviour Group, Wageningen University, Wageningen, The Netherlands; University of Akron, UNITED STATES

## Abstract

At large attitudes are built on earlier experience with the attitude object. If earlier experiences are not available, as is the case for unfamiliar attitude objects such as new technologies, no stored evaluations exist. Yet, people are still somehow able to construct attitudes on the spot. Depending on the familiarity of the attitude object, attitudes may find their basis more in affect or cognition. The current paper investigates differences in reliance on affect or cognition in attitude formation toward familiar and unfamiliar realistic attitude objects. In addition, individual differences in reliance on affect (high faith in intuition) or cognition (high need for cognition) are taken into account. In an experimental survey among Dutch consumers (*N* = 1870), we show that, for unfamiliar realistic attitude objects, people rely more on affect than cognition. For familiar attitude objects where both affective and cognitive evaluations are available, high need for cognition leads to more reliance on cognition, and high faith in intuition leads to more reliance on affect, reflecting the influence of individually preferred thinking style. For people with high need for cognition, cognition has a higher influence on overall attitude for both familiar and unfamiliar realistic attitude objects. On the other hand, affect is important for people with high faith in intuition for both familiar and unfamiliar attitude objects and for people with low faith in intuition for unfamiliar attitude objects; this shows that preferred thinking style is less influential for unfamiliar objects. By comparing attitude formation for familiar and unfamiliar realistic attitude objects, this research contributes to understanding situations in which affect or cognition is the better predictor of overall attitudes.

## Affect and Cognition in Attitude Formation toward Familiar and Unfamiliar Attitude Objects

Attitudes are built on earlier experience and help people to make sense of their environment [1]. As such, attitudes play a central role in life and make up a large part of our daily thoughts, emotions, and behavioural processes [2]. If earlier experiences are not available, as is the case for unfamiliar attitude objects, people still construct attitudes on the spot in order to respond to unfamiliar situations or attitude objects [3]. While attitudes are formed for both familiar and unfamiliar objects, the way in which these attitudes are formed can be different. The current paper investigates differences between attitude formation toward familiar and unfamiliar realistic attitude objects.

Individuals confronted with familiar attitude objects have stored evaluations in memory. If we consider attitudes as summary object-evaluation linkages and/or object-feature-evaluation linkages [4] that find their basis in cognitive and affective associations in memory [1, 5, 6], there are extensive object-evaluation linkages available for familiar objects. After individuals have been repeatedly exposed to attitude objects, additional exposures have reinforced such associations through a process of conditioning, especially if accumulating evidence has been consistent with prior knowledge [7, 8]. This results in a stable, crystallised attitude [3] that can draw upon learned cognitions such as facts and statements [9] but also in learned emotional responses such as somatic markers that are associated with past outcomes [10] and learned regulation of emotion [11]. Having an established knowledge base, consisting of relevant affective and cognitive information and experience, allows the formation of attitudes based on both cognition and affect [9, 12].

Individuals confronted with unfamiliar attitude objects, however, do not have a fully fledged evaluation of the object stored in memory. People are, nevertheless, somehow able to construct attitudes toward unfamiliar attitudes on the spot, based on whatever relevant associations are available in the current context [1, 3, 13, 14]. This raises the question what object-feature linkages will become available while constructing attitudes toward unfamiliar attitude objects. It is consistently found that, after repeated exposure to a stimulus, people will begin to react more positively to a once-novel attitude object, even without conscious evaluation [14–16]. This provides evidence that affective responses happen more quickly and possibly do not even require cognitive evaluations of attitude objects. In this way, affective object-feature linkages can rapidly emerge toward unfamiliar attitude objects. In realistic contexts, new attitude objects will rarely exist in complete isolation from anything encountered before, since attitude objects generally do not exist independently of social processes and other contextual factors [17]. Hence, in a realistic context, people will have been exposed (unconsciously or otherwise) to some features around the unfamiliar attitude object and can infer meaning from existing knowledge about related familiar attitude objects [18]. Therefore, some object-feature linkages for unfamiliar attitude objects will become activated, even if no cognitive elaboration on the new object has ever taken place. In such cases, where no cognitive inferences have yet been developed, affective responses can already be mobilised [14, 19]. This could explain why affect consistently contributes to attitude formation of unfamiliar realistic attitude objects such as, for example, carbon dioxide storage, genetic modification of food products and nanotechnology (see, for example, [20, 21]). Supporting this line of reasoning is previous research that has shown that attitudes in cases with a lack of concrete factual information are often based on affect [22, 23], which, compared to cognitive weighing of pros and cons, requires less formal information in decision-making [24]. By serving as information regarding how one feels about the attitude object, these affective associations are able to influence judgements even in the absence of crystallised cognitive or affective object-feature-evaluation linkages. In the absence of clear object-evaluation linkages, a decision based on affect is often more informative than one based on cognition, as people can more easily access the broader range of affective feelings than the few available cognitive associations [25, 26]. Hence, in situations of limited knowledge, it is more likely that people will access affective assocations toward an unfamiliar object than construct cognitive object linkages.

In this way, depending on familiarity, attitudes may find their basis in more or less elaborate affective object linkages (feelings and emotions) and/or cognitive object linkages (beliefs and thoughts) [27, 28], which represent the affective and cognitive attitude components of the overall attitude [9, 29].

Therefore our first hypothesis is:

H1: Affect will have a relatively stronger association with overall attitude for unfamiliar than for familiar attitude objects, whereas cognition will have a relatively weaker association with overall attitude for unfamiliar than for familiar attitude objects.

Although, in the process of attitude formation, people in principle have access to a rich network of cognitive and affective feature evaluations, in most daily decisions they are unlikely to access all of that information. If the object is familiar, that is in cases where both cognitive and affective object-evaluation linkages are available, individuals are at liberty to use any combination of affective object-evaluation or cognitive object-evaluation linkages that leads to a clear evaluation. Differences in individual preferences for affect or cognition will play an important role in determining to what extent people rely more on affect or cognition in attitude formation in this type of situation. Some people have a high versus low need for cognition and/or high versus low faith in intuition, reflecting differences in reliance on rational cognitive resources and affective intuitive resources. People with a high need for cognition rely on the cognitive system, making cognitive beliefs important in forming judgements [30–32], while people with a high faith in intuition rely on the intuitive system and show a strong reliance on feelings in their judgements [31–33]. People with a clear preferred thinking style (high need for cognition and low faith in intuition or vice versa) will rely on either cognition or on affect, depending on their preference. For instance, people with a high faith in intuition seek out affective information while forming attitudes and are more strongly persuaded by affective messages. In the same way people with a high need for cognition, look for cognitive information while forming attitudes [34]. In this way chronic individual differences are important determinants why attitudes are more affect-based or cognition-based [35].

If the object is unfamiliar, however, cognitive object-evaluation linkages are hardly available, while affective-evaluation linkages remain to a larger extent available. For unfamiliar attitude objects, individuals are restricted in relying on cognitive evaluations and therefore can only fall back on more intuitive and affective processes. Thus, even when individuals have a high need for cognition, an analytical approach to information processing might fall short for unfamiliar objects [36]. It is therefore expected that, for unfamiliar attitude objects, a preference for a cognitive thinking style will not relevantly shift the process to a more cognition-driven process. As affect is less dependent on information, affect will have a similarly high influence on the overall attitude for both familiar and unfamiliar attitude objects, for people having a high faith in intuition. A high influence of affect on overall attitude is expected even in the case of low faith in intuition for unfamiliar attitude objects. Thus, for unfamiliar attitude objects individual differences might not always be important determinants why attitudes are more affect-based or cognition-based.

Hence, we hypothesise that:

H2a: For familiar attitude objects cognition will have a relatively stronger association with overall attitude for individuals with high need for cognition, compared with low need for cognition.
H2b: For unfamiliar attitude objects, cognition will not have a relatively stronger association with overall attitude for individuals with high need for cognition, compared with low need for cognition.
H3a: For familiar attitude objects affect will have a relatively stronger association with overall attitude for individuals with high faith in intuition, compared with low faith in intuition.
H3b: For unfamiliar attitude objects affect will have a relatively stronger association with overall attitude for individuals with both high and low faith in intuition.

## Study

The hypotheses were tested in an experimental survey in the Netherlands in the context of technological applications. Nanotechnology, an existing yet little known novel technology, was used to study a range of unfamiliar attitude objects. The range of unfamiliar nanotechnology attitude objects was compared comparing to a similar range of familiar variants. Nanotechnology provides a good research context to operationalise unfamiliar realistic attitude objects as, at the time of the study, people had limited knowledge about nanotechnology and its applications [37]. Nanotechnology is involved with the manipulation of materials at the smallest possible physical levels (i.e. molecular or atomic levels). It enables the creation of completely new products, as well as the substantial improvement of properties of existing products [38]. By focusing on nanotechnology-facilitated improvement of existing products, nanotechnology not only provides a good research context to operationalise realistic unfamiliar attitude objects but also allows for comparison between similar unfamiliar and familiar attitude objects. Nanotechnology also allows us to select from a broad range of applications, which may be associated with different potential advantages and disadvantages, helping to show robustness of results.

## Method

### Respondents

Data were collected by a commercial market research agency (GfK; see www.gfk.com), as the first wave in a longitudinal study. Respondents were drawn from an existing panel for which respondents voluntarily registered. The research complies with the Netherlands Code of Conduct for Scientific Practice and the Social Sciences Ethics Committee of Wageningen University waived the need for ethical consent. There were no misleading questions in the survey, and the questions did not cause discomfort to respondents. The participants opted into this study that was conducted online through the market research agency GfK. The authors did not have access to any identifying information about the participants as GfK anonymized and de-identified all data prior to author access.

As the socio-demographic information of panel members is known to the research agency, the panel allows for stratified random sampling of a nationally representative sample on gender, age, and education level of the Netherlands. The panel consists of approximately 12,000 respondents, who are repeatedly invited to participate in studies. The panel is maintained through a range of sampling techniques, taking care that the panel remains representative for the population. Agreement to join the panel is between 10% and 35% among those invited; about 20% of panel members are replaced each year. Data were collected in the Netherlands between 16 October and 6 November 2012. The research agency approached a gross sample of 2500 respondents from their panel, of whom 1907 participated (response rate of 76%). Of these, 51% were male, 28% have a low education level (primary school, vocational education), 45% have an intermediate (secondary vocational education), and 27% have a high education level (university of applied sciences, or university). The mean age of respondents was 43 years (SD = 13.2 years, age range 18–65 years).

Inspection of responses on all variables showed highly unlikely response patterns for 37 respondents, who had zero variance on all variables. These 37 respondents were removed prior to analyses (valid *N* = 1870). After removing these respondents, no further univariate and multivariate outliers were detected.

### Design

Respondents judged a total of four applications. Each respondent was asked to rate both familiar (non-nanotechnology) applications and the related unfamiliar (nanotechnology) applications. In addition, each respondent was asked to judge applications from two out of four application domains (either water and energy, or medicine and food). Within each application domain, two different applications were specified. Each respondent judged in total four of the sixteen available applications, as an incomplete repeated measures factor across four domains. The combinations of stimuli as presented to groups of respondents can be found in Table 1.

**Table 1:** Design of the study.

Group	N	Nano application	Non-nano application	Nano application	Non-nano application	Nano application	Non-nano application	Nano application	Non-nano application
1	449	Water purification nano-membrane	Water purification sand filtration	Nano-solarcel	Solarcel				
2	471	Water monitoring nano sensor	Water monitoring	Nano battery	Battery				
3	495					Lab-on-a-chip	Medical home test	Nano food additives	Food additives
4	492					Nano medicine	Medicine	Nano supplements	Supplements

Note. This is the first study in a longitudinal series.

### Stimuli

#### Technological applications

Stimuli were 16 vignettes of technological applications. These consisted of a set of eight familiar and eight unfamiliar (nano) applications with the same purpose to operationalise different levels of familiarity. For example, a conventional solar panel was included as a familiar attitude object and a nano-based solar panel was included as an unfamiliar attitude object. Four application domains were included that cover the key areas of nanotechnology research and development: food, water purification, medicine, and energy [39]. For each domain, two different applications were selected—food additives and food supplements, water purification and water quality monitoring, medical home tests and drugs, solar energy and batteries—to provide replications, allowing us to control for specific application and domain associations.

Respondents received a short description of an application, consisting of some information about the technology behind the application, examples in which the application can be used, and some advantages and disadvantages of the application. The order of the information on advantages and disadvantages was randomised. Descriptions were checked by an expert on nanotechnology for realism of content. Scenarios of pairs of applications (familiar versus unfamiliar) were matched as much as possible in content and length. An example of a scenario is provided in File A in S1 appendix (translated from Dutch).

Four sequential pilot studies were conducted to improve the materials iteratively. The first three pilot studies were conducted with students from Wageningen University, and the final pilot study was conducted on a more diversified sample. Scenarios were adapted until comprehensibility, credibility, and emotional neutrality, as well as the purpose, advantage, and disadvantage of the familiar and unfamiliar applications, were comparable; at the same time, the nano applications remained less familiar compared to corresponding alternatives (see Table A in S1 appendix for details).

### Measures

Respondents were asked whether they had heard of nanotechnology (‘yes/no’) and whether they knew what nanotechnology means (‘yes/no’). To assess whether familiarity with the selected applications differed between the nano and the conventional applications, respondents were asked to indicate for each application whether they had heard of the application.

Affective attitude. The affective attitude component was measured with an affective judgement scale, consisting of four items measuring positive emotions (joy, desire, fascination, satisfaction) and four items measuring negative emotions (fear, boredom, sadness, and disgust) (see [40, 41]). Respondents were asked to indicate to what degree they experienced each of the emotions when reading about the application, on a seven-point Likert scale (1 = ‘not at all’, 7 = ‘very much’).

Cognitive attitude. The cognitive attitude component was measured with a cognitive judgement scale, consisting of four items measuring positive cognition (useful, functional, beneficial, nice) and four items measuring negative cognition (useless, harmful, disadvantageous, unusable) (based on [42]). Respondents were asked to indicate to what degree they think each of the cognitions applied when reading about the application on a seven-point Likert scale (ranging from ‘not at all’ to ‘very much’).

#### Scale dimensions

In order to verify that the cognitive and affective items tapped into different attitude components, a hierarchical confirmatory factor analysis (CFA) was conducted with maximum likelihood in the R package Lavaan [43]. Comparative fit index (CFI) and Tucker Lewis Index (TLI) values above .95, and root mean square error of estimation (RMSEA) values below .07 were adopted as indication of good fit. *χ²* is reported as customary, but not indicative of model fit with large samples [44]. Confirmatory factor analyses (CFA) were conducted on all key concepts (affect, cognition, overall attitude) taking into account the negative versus positive wording of items [45]. After removal of the ‘boredom’ (affective negative) and ‘nice’ (cognitive positive) items, which did not load onto their respective factor, the CFA showed a good fit χ² (63) = 1476.74, p <. 001; CFI = .98; TLI = .97; and RMSEA = .055 [.052 to .057]. Items were then recoded where needed and averaged to form reliable affective (Cronbach’s alpha = .78; *M* = 4.83, SD = 1.07) and cognitive (Cronbach’s alpha = .86; *M* = 4.45, SD = 1.01) attitude scales.

Overall attitude. Overall attitude was measured with one item: ‘What is your overall opinion toward the application?’, measured on a seven-point scale (1 = ‘very negative’ and 7 = ‘very positive’). Single item constructs can be considered a relevant replacement for multi-item constructs in many cases [46–47]. For the current study, the choice for a single-item attitude scale was validated in another study where attitude was measured on a multi-item scale (including the item used here) and the same affect and cognition scales that showed comparable *b*’s for the single-item measure (.58 for affect and .52 for cognition) and the multiple-item measure (.58 for affect and .55 for cognition) (details available from authors upon request).

#### Need for cognition and faith in intuition

Respondents’ need for cognition and faith in intuition were measured using a Dutch translation of the short version of the Cognitive-Experiential Inventory (for the REI, see [31]). After recoding negative items, internal scale reliability of the need for cognition scale and faith in intuition scale were high (Cronbach’s alpha = .77 and .88, respectively). There is a negligible correlation between need for cognition and faith in intuition, *r* (1873) = .046, *p* < .001, and a moderate correlation between education level and need for cognition, *r* (1873) = .328, *p* < .001. The mean for need for cognition was 4.93 and for faith in intuition 4.61 (measured on 7-point scales). Familiarity and need for cognition were somewhat correlated (r(1873) = -.156, p < .001), in such a way that higher need for cognition relates to higher familiarity with nanotechnology.

### Procedure

Respondents started the online survey in their own home at their own time and were presented with an introduction to the study. Next, all respondents answered two questions about their knowledge about nanotechnology in general, before reading a general description of nanotechnology in order to create a basic understanding about nanotechnology. Respondents were then randomly assigned to one of four conditions (see Table 1). After reading the general description, respondents were asked to carefully read an information scenario of one of the four applications. After they read the information, respondents’ affective attitude, cognitive attitude, overall attitude, and familiarity with the application were measured. This process was repeated for all four applications in randomised order. At the end of the survey, respondents were asked to complete the REI questionnaire and to provide some other background variables. As this study was part of a larger study, additional background information collected for purpose of the larger study was not used in the analyses. The following were measured: subjective cognitive and affective ambivalence; personal and societal relevance nanotechnology; perceived risks and benefits; trust about regulations and governance; attitude toward nanotechnology in general; devotion to science; and media use. None of these covariates were included in further analyses. Demographic information was available through the panel registration.

Respondents were thanked for their participation and debriefed. In the debriefing, the respondents were told that the majority of nanotechnology applications used for the survey are still under development and therefore non-existent at this time. Following their participation, the respondents received credits from the research agency that could be accumulated toward a gift voucher. On average, it took about 22 minutes to complete the questionnaire.

### Data analysis

To assess the impact of *affect* and *cognition*, *familiarity* of the technology/application, and *need for cognition* and *faith in intuition* on *overall attitude*, attitude scores were subjected to a repeated-measures mixed linear model using SPSS 19. Mixed linear models can deal with incomplete repeated measures (respondents rated four out of sixteen applications). *Application* was entered as a repeated variable in the model. A simple-structure variance–covariance matrix was set. Familiarity was operationalised as the difference between nano applications (unfamiliar) and conventional applications (familiar). Familiarity with the technology was effect coded (familiar = –1; unfamiliar = 1). In addition to the variables of interest, the eight application types were included in the model as effect-coded covariates to control for associations with the specific application. Scores on the continuous independent variables (affective and cognitive attitude, need for cognition and faith in intuition) were grand mean centred to control for collinearity between main effects and (un-centred) interaction effects [48].

A model was estimated with all main effects and the two- and three-way interactions of interest. Unstandardised betas are reported in the results. To interpret three-way interaction effects, simple slope analysis was used. Simple slope analyses are used to illustrate the interaction effect, with one standard deviation below the mean and one above the mean of the predictor (see [49]). Effect sizes for mixed linear models were not readily available at the time of writing. The survey data are available in S1 dataset.

## Results

While 71.9% of respondents recalled having heard of the term nanotechnology, only 39% of respondents reported knowing its meaning. Thus, in general, knowledge about nanotechnology is low. In addition, the proportion of respondents indicating knowledge about the selected nanotechnology applications was lower (27%) than the proportion of respondents indicating knowledge about their conventional counterpart (55%), *χ²* (7) = 93.71, *p* <. 05, Cramer’s *V* = .12; this confirmed the successful operationalisation of familiarity by presenting respondents with conventional versus nano-based applications.

### Model tests

Affect, cognition, familiarity. The results show that affect and cognition both have a positive main effect on the overall attitude, *F*_affect_ (1, 7473) = 1594.73, *p* < .001, *F*_cognition_ (1, 7473) = 1359.27, *p* < .001 (see Table 2 for details). The regression coefficient of affect is .59 and of cognition .52. Familiarity has a positive main effect on overall attitude, *F* (1, 7473) = 9.65, *p* = .002. The interaction between familiarity and affect has an effect on the overall attitude, *F* (1, 7473) = 9.47, *p* = .002, in such a way that affect has a higher association with overall attitude toward unfamiliar technologies than familiar technologies. The interaction between familiarity and cognition has an effect on overall attitude, *F* (1, 7473) = 7.02, *p* = .008, showing that cognition has a higher association with overall attitude toward familiar technologies than unfamiliar technologies. Thus, as predicted in hypothesis 1, there is a relatively stronger association between affect and overall attitude for unfamiliar attitude objects and a relatively stronger association between cognition and overall attitude for familiar attitude objects.

**Table 2:** Results of general linear mixed model analysis.

	Variable	*t*	*b*	*p*	*CI95* lower	*CI95* upper
H1	Affect	39.93	.585	.00***	.557	.614
	Cognition	36.87	.518	.00***	.490	.545
	Familiarity (familiar = -1; unfamiliar = 1)	-3.11	-.035	.002**	-.057	-.013
	Familiarity*affect	3.08	.045	.002**	.016	.073
	Familiarity*cognition	-2.65	-.037	.008**	-.064	-.010
H2	Need for cognition (nCog)	-4.42	-.048	.00***	-.068	-.027
	nCog*affect	-1.01	-.015	.31	-.043	.014
	nCog*cognition	4.70	.065	.00***	.038	.093
	nCog*familiarity	.75	.008	.46	-.013	.029
	nCog* familiarity*affect	1.25	.018	.21	-.010	.047
	nCog*familiarity*cognition	-1.51	-.021	.13	-.048	.006
H3	Faith in intuition (FI)	4.09	.040	.00***	.020	.059
	FI*affect	2.37	.030	.02*	.005	.056
	FI*cognition	-3.25	-.040	.001**	-.065	-.016
	FI*familiarity	.43	.004	.67	-.015	.024
	FI*familiarity*affect	-2.15	-.028	.03*	-.054	-.003
	FI* familiarity*cognition	2.51	.031	.01*	.007	.056

Note.

* < .05;

** < .01;

*** < .001.

Application type was controlled for and entered as covariate, F (7, 7466) = 21.27, p < .001.

Need for cognition. Need for cognition had a negative main effect on overall attitude, *F* (1, 7473) = 19.56, *p* < .001. In addition, the significant two-way interaction between need for cognition and cognition shows that higher need for cognition has stronger associations with cognition on overall attitudes, *F* (1, 7473) = 22.11, *p* = .006. The non-significant three-way interaction of need for cognition, cognition, and familiarity shows that this effect is similar for familiar and unfamiliar attitude objects (see Table 2). These results confirm that there are stronger associations between cognition and overall attitude for high need for cognition for familiar attitude objects, in line with H2a. However, this is also the case for unfamiliar attitude objects, and not only for familiar objects, showing no support for H2b. The same pattern is reflected in the simple slope analyses, which show that cognition has a stronger association with the overall attitude for high need for cognition compared with low need for cognition, for familiar and unfamiliar attitude objects (see Table 3).

**Table 3:** Regression coefficients nCog and FI^1^ (H2; H3) for familiar and unfamiliar applications, based on simple slope analyses.

Variable	Familiar^2^		Unfamiliar^3^	
	Affect	Cognition	Affect	Cognition
High nCog	.51	.63	.62	.52
Low nCog	.57	.47	.62	.43
High FI	.60	.48	.63	.46
Low FI	.48	.62	.62	.48

Note.

^1^ nCog is the abbreviation of need for cognition; FI for faith in intuition.

^2^ Baseline for familiar: regression coefficient affect = .54; regression coefficient cognition = .55.

^3^ Baseline for unfamiliar: regression coefficient affect = .63; regression coefficient cognition = .47. High and low relate to simple slope analyses centring that variable at one SD above the mean and one SD below the mean; for significance levels, see Table 2 (all < .05).

The influence of need for cognition on affect was investigated and showed no significant interaction between need for cognition and affect. In addition, there is no significant three-way interaction between familiarity, need for cognition, and affect. Thus, as expected, need for cognition does not influence the associations between affect and overall attitude.

Faith in intuition. Faith in intuition has a positive effect on overall attitude, *F* (1, 7473) = 16.75, *p* < .001. In addition, the interaction between faith in intuition and affect shows that higher faith in intuition creates stronger associations between affect and overall attitude, *F* (1, 7473) = 5.62, *p* = .018. More importantly, in line with hypothesis 3, the interaction between familiarity, faith in intuition, and affect on overall attitude is significant, *F* (1, 7473) = 4.64, *p* = .031. Simple slope analyses show that affect has a stronger association with the overall attitude with high faith in intuition compared with low faith in intuition for familiar attitude objects in line with H3a. In addition, it is shown that high and low faith in intuition do not influence the importance of affect for unfamiliar attitude objects in line with H3b (see Table 3).

The influence of faith in intuition on cognition shows a significant interaction between faith in intuition and cognition, *F* (1, 7473) = 10.57, *p* < .001. In addition there was a significant three-way interaction between familiarity, faith in intuition, and cognition on overall attitude, *F* (1, 7473) = 6.30, *p* = .012. Simple slope analyses show that cognition has a stronger association on the overall attitude for people with low faith in intuition compared to high faith in intuition for familiar attitude objects (see Table 3). Faith in intuition does not alter the influence of cognition for unfamiliar attitude objects. Thus, for familiar attitude objects only, for people with high faith in intuition affect has a stronger association with the overall attitude and at the same time the association of cognition with the overall attitude is reduced.

## General Discussion

This study showed that attitude formation processes for realistic unfamiliar attitude objects rely more on affect than is the case for realistic familiar attitude objects. By focusing on unfamiliar realistic attitude objects, where some knowledge in the context can be expected, the current study addresses the gap between attitude research that either focused on attitude objects where a meaningful reference point is lacking (e.g. fictitious attitude objects) or focused on familiar attitude objects. The present study achieved this by presenting similar applications with and without the use of a new unfamiliar technology: nanotechnology.

The results showed that, for the more familiar attitude objects, cognition is more predictive for the overall attitude. On the other hand, for realistic unfamiliar attitude objects, affect is more predictive for the overall attitude. This supports the proposition that, in attitude formation toward familiar attitude objects, people in principle have access to a rich network of affective and cognitive associations and feature evaluations. For familiar attitude objects, evaluation (the extent to which people rely on affect or cognition) is guided by people’s preferred thinking style. Consistent with previous research, people with high need for cognition rely more on cognition in attitude formation and people with high faith in intuition rely more on affect [31, 34, 35].

The current study shows that people with high need for cognition rely more on the influence of cognition on overall attitude, both for familiar and also (contrary to the expectations) for unfamiliar attitude objects. Previous research showed that, for unfamiliar attitude objects without meaningful reference points, repeated exposure leads to positive affective feelings in the lack of a solid knowledge base [14, 16]. Our study shows that, with realistic unfamiliar attitude objects, cognitions can be constructed or derived from the realistic context if individuals have a high need for cognition. Thus, cognitions can be used to some extent toward unfamiliar attitude objects. A possible explanation for this, which should be further investigated, could be that individuals in the current study with a high need for cognition also had higher education levels and are therefore more familiar with nanotechnology and have a better comprehension of nanotechnology, leading to attitudes that are based more on factual (cognitive) information.

The current study extends previous research on faith in intuition in relation to affective focus for unfamiliar attitude objects. For unfamiliar attitude objects, both high- and low-intuitive people rely on affect, while for familiar attitude objects in particular, people with high faith in intuition rely on affect. This implies that, for people with low faith in intuition, affect can still be considered as a default heuristic [50], making best sense of unfamiliar realistic attitude objects.

We presented respondents with unfamiliar realistic attitudes delivering potential benefits in an unfamiliar way (based on nanotechnology). As the selected attitude object is a technology application, and the unfamiliar variant is a similar application based on a novel technology, technophobia or consumer resistance against technology may play a role [51]. Specifically, with new technologies, ‘unknown’ can quickly turn into ‘unloved’, as happened for instance in the case of genetically modified food [52]. Yet, as the technology context brings in specific characteristics for nanotechnology, most will also be applicable for their conventional alternatives. In addition, attitude objects in a technology context are more utilitarian in nature, which might trigger more cognitively based attitudes. It is however also the case that for unfamiliar attitude objects in a technology context attitudes are more strongly based in affect. Nevertheless, future research should focus on other unfamiliar attitude objects that deliver familiar benefits in an unfamiliar way (for instance when smartphones just came onto the market). Hence, research should be extended to include different unfamiliar objects to generalise these findings and to control for specific context-dependent heuristics.

Respondents were provided descriptions about the attitude objects in order to make any sense of the stimuli. Careful pilot testing confirmed that the scenarios were emotionally neutral. The elaborateness of information of the scenarios may however have contributed to a factual basis, necessary to construct cognitive attitudes, and thus may have reduced hypothesised effects that were nevertheless observed. Providing a short scenario only once will do little to support the creation of lasting cognitive or affective associations. Research following longitudinal exposure to the technology in real-life context would be a more relevant way to study familiarisation of the new technology. In addition, in future research it is also important to investigate whether the relative influence of affect and cognition on overall attitudes hold when the available context information changes. For instance, when there is little or no information about unfamiliar objects presented, as is often the case in real life.

Attitudes were assessed in a representative adult sample of the Netherlands (i.e. as opposed to a student sample), less familiar with technological innovations. This supports the predictive and explanatory value of the cognitive–affective attitude structure for the general population. A disadvantage of survey research is however that it is not possible to disentangle the underlying processes in attitude formation [53] or biases observed with self-report measures [54]. In order to get a deeper understanding of how affective and cognitive processes influence the attitude formation process, additional methods that do not rely (completely or partially) on self-reports should be used in future research. Techniques such as time-pressured answering or psychophysiological measures, such as heart rate variability, galvanic skin response, eye-tracking, or fMRI, extend the possibility to study the actual processes without interfering in the research context, and provide deeper insights about underlying processes [55]. It is pragmatically impossible to apply these to large representative samples; hence, a combination of large-scale population-based surveys, with focused experiments to further understand the underlying processes, is recommended for future research.

As a final remark, it should be emphasised that communication toward the general public is often cognitive in nature, with a focus explaining and rationalising new things and innovations [56]. The present study shows that it is important (or even essential) to anticipate emotions and address people’s affect in communication toward the general public. This study shows that, in order to understand how the public will respond to real-life innovations, in-depth understanding of the formation of attitudes and of the balance of affect and cognition toward unfamiliar but realistic attitude objects is essential. While cognition plays a role in attitude formation toward unfamiliar realistic attitude objects when people have a high need for cognition, in general, affect is the more influential predictor of attitudes toward unfamiliar realistic objects.

## Supporting Information

S1 Appendix File A in S1 Appendix provides an example of a scenario used in the current study. Table A in S1 Appendix provides detailed analyses on the pilot studies.
(DOCX)

S1 Dataset This file provides the dataset (sav / SPSS file). (SAV)
