# Supplementary material for: Affect and Cognition in Attitude Formation toward Familiar and Unfamiliar Attitude Objects
Source: PLoS One. 2015 Oct 30;10(10):e0141790. doi: 10.1371/journal.pone.0141790 (PMC4627771; doi:10.1371/journal.pone.0141790)
Supplement: S1 Appendix — Table A in S1 Appendix provides detailed analyses on the pilot studies. (DOCX) [file pone.0141790.s001.docx]

# File A

Example scenarios (translated from Dutch*)*

### Scenario 1: Water filtration using a nano-membrane

**Water purification and desalination by means of ultra-fine nano-membranes**

The water industry is working on the development of ultra-fine membranes with nano-materials for water purification. This membrane acts like a coffee filter for molecules, ensuring that water is purified for drinking. Certain substances can pass through the membrane and other substances are stopped. An example is the desalination and disinfection of water for drinking.

Because the nanoparticles in the membrane change the properties of the membrane, they ensure that the membrane draws in water. As a result, the water can easily go through the membrane. With the same amount of pressure, twice as much water can be purified. The nano-membranes can therefore increase the efficiency of purification and desalination of water for drinking.

People use water and can therefore be exposed to released nanoparticles. For instance, some of the nanoparticles can penetrate into the brain and into cells, where larger particles cannot pervade. It is not yet known what the consequences are to humans. The nanoparticles could potentially cause damage.

### Scenario 2: Water filtration using sand filtration

**Water purification by means of sand filtration**

In the water sector, sand filtration is used for water purification. With sand filtration, the water flows through a bed of fine sand and/or gravel. When water flows through this filter, dirty particles in the water are retained. Water itself can flow through the filter. An example is removing waste from water for drinking or deferrisation of groundwater by sand filtration.

Because a sand filter is easy to install, sand filtration can be easily used in many places in water-management systems. Sand filters can be used as pre-treatment and post-treatment methods. Thus, water can be purified efficiently and cheaply.

People use water for instance for showering and drinking. Sand filtration is not a total disinfection method. Thus, nematodes and viruses are in some cases insufficiently filtered, so that they can enter the body. People could be exposed to these nematodes and viruses and consequently become ill.

# Table A. Description of pilot studies

|  | Pilot study 1 | Pilot study 2 | Pilot study 3 | Pilot study 4 |
| --- | --- | --- | --- | --- |
| Aim | Investigate comprehensibility, credibility, completeness. | Compare familiar and unfamiliar applications on the comparability of the purpose of the technology, advantage, and disadvantage.  Feedback from respondents to improve scenarios. | Compare standalone text of either purpose, advantage, or disadvantage of technology on comprehensibility, credibility, complexity, emotional neutrality. | To confirm that the questionnaire is comprehensible for respondents other than university students; to find flaws in scales and survey. |
| Sample | Wageningen University students (*N* = 18, *M*_age_ = 23.67 years, SD_age_ = 2.61). | Wageningen University students and staff (*N* = 20; *N*_per application_ = 10). | Wageningen University students (*N* = 80; *N*_per text_ = 10). | Sample with different backgrounds and ages (*N* = 21; *N*_per application_ = 5; *M*_age_ = 35, SD_age_ = 14.84). |
| Stimulus material | Short description of purpose of application. | Adjusted scenarios with description of purpose, advantage, and disadvantage of the technology. One for the familiar technology and one for unfamiliar technology, which needed to be compared. | Six texts of different technologies, with only the isolated purpose, advantage or disadvantage. | Final scenarios (of which an example is presented in S1). |
| Measures | Seven-point Likert scales (not at all – very much):  *Comprehensibility  *Credibility  *Completeness | (1) Seven-point Likert scales (not at all – very much):  *Comparability of purpose  *Comparability of advantage  *Comparability of disadvantage  (2) Remarks of respondents | Seven-point Likert scales (not at all – very much):  *Comprehensibility  *Credibility  *Complexity  *Emotional neutrality | (1) Seven-point Likert scales (not at all – very much):  *Comprehensibility  *Credibility  *Complexity  *Clarity  (2) Questionnaire as used in the study |
| Results | Since this is the first pilot study in an iterative set of studies, it does not make sense to report all results from the first studies. In general, some applications were more comprehensible and credible than others. Overall, all scenarios were seen as incomplete. | One sample t-tests showed:  *Comparability of purpose, *t* (7) = -.023, *P* = non significant (ns).  *Comparability of advantage, *t* (7) = .197, *P* = ns.  *Comparability of disadvantage, *t* (7) = .009, *P* = ns.  Medicine and supplements had relatively low means and were adjusted and piloted for a second time. | For the aggregated applications, a MANOVA shows that all applications were considered equally emotionally neutral (*F* (1, 3) = .38 *p*_purpose_= 1.0; *p*_advantage_ = .60; *p*_disadvantage_ = .29). | *Comprehensibility: all means >5  *Credibility: all means > 5.3  *Complexity: all means > 5; except lab on a chip and medical test. These scenarios were improved afterwards  *Clarity: all means > 5  *Knowledge familiar (81%) > Knowledge unfamiliar (45%)  *Affect scale: *α* = .77  *Cognition scale: *α* = .75 |
| Main conclusions | Scenarios were credible; however, not considered complete. | Scenarios of familiar and unfamiliar technology were in content comparable. Thus a similar purpose, advantage, and disadvantage were described. | Pairs of familiar and unfamiliar applications are comparable on all criteria. | All scenarios were considered as comprehensible, credible, understandable, and clear. Familiar technologies were seen as more familiar than unfamiliar technologies. |
| **Main conclusion:** With confidence we can conclude that stimulus material does not differ in comprehensibility and emotional neutrality. Furthermore, familiar technological applications were judged as more familiar than unfamiliar applications. | | | | |
